# Bilgewater management in marine vessels: a systematic literature review of marine vessel bilgewater and treatment options

**DOI:** 10.1007/s11356-026-37673-4

**Published:** 2026-03-27

**Authors:** Jason Leonard, Mayisha Ahmedullah, Melissa Brown, Ingrid Brundin, Howard Fallowfield, Mikael Johansson, Claire Lenehan, Sophie Leterme, Scott Wade, Matthew Yuen, Harriet Whiley

**Affiliations:** 1 ASC Pty Ltd, Adelaide, Australia; 2 College of Science and Engineering, Flinders University, Adelaide, Australia; 3 ARC Training Centre for Biofilm Research and Innovation, Flinders University, Adelaide, Australia; 4 School of Engineering, Swinburne University, Melbourne, Australia; 5 Flinders Factory of the Future, Adelaide, Australia

**Keywords:** Bilgewater, MARPOL Annex I, Marine Environmental Protection, Marine biosecurity, Biodegradation

## Abstract

Bilgewater is a mixture of oil, water, suspended solids, and other contaminants that collects in the lower inside areas of marine vessels and its discharge into the marine environment contributes to ocean pollution. Due to its complex composition and ecological impacts, effective management is critical. This systematic review examined existing research on bilgewater composition and assesses available treatment technologies to identify knowledge gaps and options for treatment. The Scopus database was searched for studies published between 1980 and 2025 that examined bilgewater treatment or its chemical and microbiological characteristics. Studies lacking independent validation or functioning as commercial promotion were excluded to minimise bias. A total of seventy-seven studies were examined. These revealed significant heterogeneity in bilgewater’s chemical and microbiological profiles across vessels, sampling locations, and environmental conditions. Microbiological analysis identified the presence of hydrocarbon-degrading bacteria, corrosion-associated species, and potentially pathogenic species. Bilgewater treatment approaches included biological, electrochemical, filtration, coagulation, and multi-barrier systems, each with distinct advantages and limitations. The variability of bilgewater composition highlights the need for standardised assessment methods and guidance on which treatment systems are most effective in different situations. Future research is also needed to understand the interplay between the chemical and microbial characteristics of bilgewater and to investigate the removal of trace metals, non-indigenous species, pathogenic bacteria, and emulsified oil. Addressing these challenges is imperative to developing robust regulations and innovative control solutions.

**Graphical abstract:**

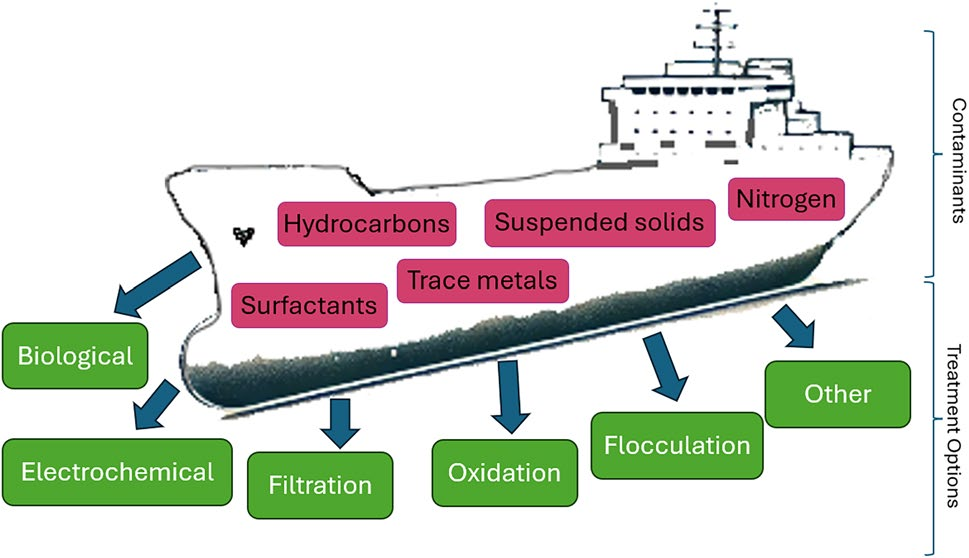

**Supplementary Information:**

The online version contains supplementary material available at 10.1007/s11356-026-37673-4.

## Introduction

Oceans are fundamental to global health, providing crucial ecosystem services like climate regulation, nutrient cycling, and biodiversity support (Miloslavich et al. 2018; Worm et al. 2006). The health of these ecosystems directly influences human well-being by determining the availability and quality of ecosystem services, which are essential for sustaining human societies and the broader natural world (Hernández‐Blanco et al. 2022). Ocean pollution therefore is a significant issue that threatens marine resources, ecosystems, and human health. Maritime discharges from ship bilgewater are a source of ocean pollution that has been receiving increasing scrutiny (Evanisko 2020; Riadh 2024). Hydrocarbons and heavy metals, which are significant constituents of ship discharges **(**Tiselius and Magnusson 2017**)**, are known to pose severe risks to marine ecological integrity **(**Ansari et al. 2004**)**. The maritime industry is crucial to the global economy as shipping is responsible for 80% of global trade. In 2023, 11 billion tonnes of freight were transported by sea (UNCTAD 2023), that had risen to over 12 billion by 2024, and it is forecast to continue increasing (UNCTAD 2024). This growth in the shipping sector will also present increased environmental challenges.

Bilges are located in the lowest part of a compartment on a ship, and are designed to collect water, oil, and other fluids that drain from various parts of the vessel. Thus, bilgewater is comprised of any substance that makes its way to the bilges. Bilgewater is typically an oily water mixture that can contain a mix of salt water, freshwater, oil, hydraulic fluids, surfactants, marine species, bacteria, viruses, suspended solids, and other contaminants (Gatidou et al. 2022b; Malakhov et al. 2024).

The variability of bilgewater production in ships and the sometimes-clandestine nature of its discharge complicates the estimation of bilgewater discharge into the ocean. Modelling using the Ship Traffic Emission Assessment Model (STEAM) estimated that 288,000 tons of bilge water including an estimated 0.9 ton of oil was discharged into the Baltic sea in 2012. (Jalkanen et al. 2021). (National Academies of Sciences and Medicine 2022) estimate the global machinery bilge oil discharge in 2020 to be 270 tonnes per year. This modelling assumes full regulatory compliance and thus may underestimate the true nature of bilgewater discharge. Furthermore, with global maritime trade projected to grow by 2.4% each year to 2029 (UNCTAD 2024), it is expected that the amount of bilgewater discharge will also grow in the future.

Bilgewater is highly variable both in chemical (Medeiros et al. 2022) and microbiological composition (Corti-Monzón et al. 2021) and can be significantly different even on the same vessel at different times or in different sections of the ship (Wood et al. 2021). Microbial composition varies significantly between sessile communities, which are composed of bacteria that adhere to surfaces, and planktonic communities, which consist of bacteria that live suspended in the water column (Wood et al. 2021). This presents significant challenges in estimating the environmental impact and optimising treatment solutions to meet regulatory guidelines (Julian 2000). The international convention for the prevention of pollution from ships (MARPOL) is an agreement that was first adopted in 1973 to address marine pollution (Julian 2000). It contains six annexes which cover the different forms of pollution generated by ships. Although the IMO regulates the spread of non-indigenous species (NIS) in ballast water through its international convention for the control and management of ships’ ballast water and sediments, NIS in bilgewater remain unregulated. The transfer of NIS in bilgewater has been shown to be a concern, with studies in New Zealand, North America, and the Mediterranean showing potential for NIS transfer in bilges (Darbyson et al. 2009; Fletcher et al. 2017; Johnson et al. 2001; Maggio et al. 2023).

This systematic literature review examines studies that characterise the chemical and biological composition of bilgewater. It also investigates the advantages and disadvantages of different treatment options. This information will inform future management and regulation for the control of ocean pollution resulting from bilgewater discharge. This is of global importance given the increasing focus on environmental issues arising from marine discharge.

## Materials and methods

**Fig. 1:**
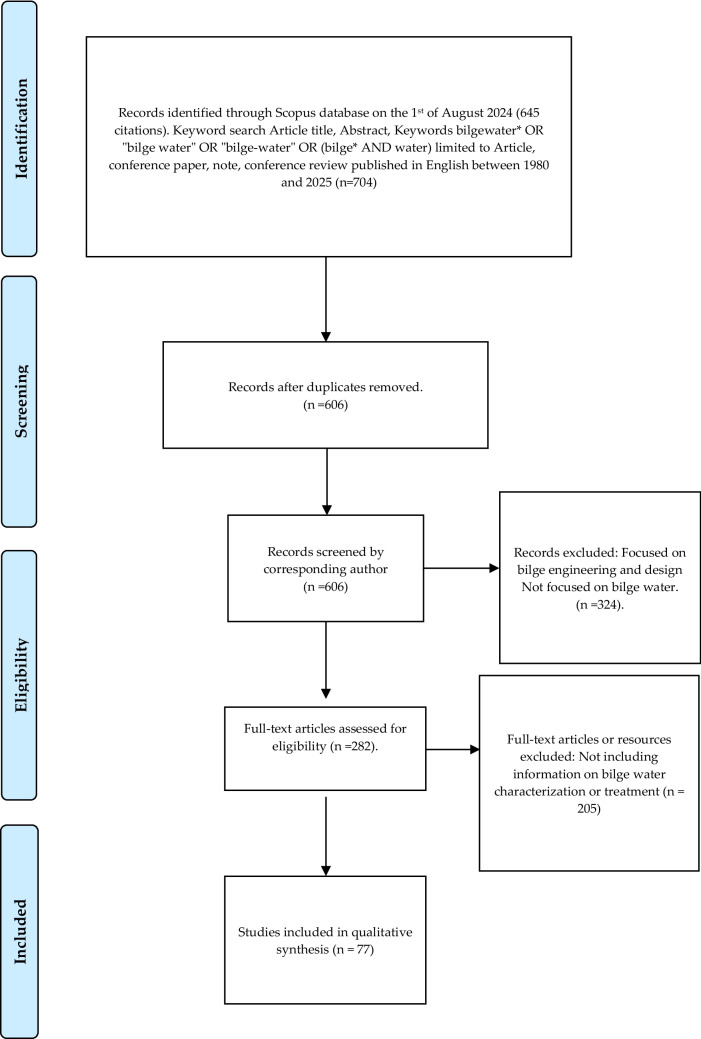
Overview of search methods and the inclusion and exclusion criteria for articles

This systematic literature review was based on a modified PRISMA (Page et al. 2021); the steps are shown in Fig. 1. Scopus was selected as the primary database due to its status as the largest abstract and citation database of peer-reviewed literature, offering comprehensive coverage across the interdisciplinary fields of Maritime Engineering, Environmental Science, and Microbiology. While Scopus provided wider journal coverage than Web of Science, PubMed was excluded due to its clinical focus, which offered limited relevance to the industrial and technical nature of bilgewater management. The Scopus database was searched on the 1 st of August 2024 for the keywords bilgewater OR “bilge water” OR (bilge AND water) appearing in the article title, abstract, or keyword. To ensure relevance to current maritime standards, the search was limited to articles published between 1980 and 2024. Results were limited to articles, conference papers, notes, or conference reviews published in English. Ninety-eight duplicates were removed, and the remaining 606 abstracts were screened for relevance. Articles were included if they investigated bilgewater treatment or the characterisation of real bilgewater samples. Any articles outside this scope, including articles related to bilge design or hydrodynamics, were excluded. The remaining 282 full text articles were assessed for eligibility using the same inclusion and exclusion criteria. To minimise commercial bias, studies identified as promotional materials or evaluations conducted solely by the manufacturer were excluded.

**Fig. 2:**
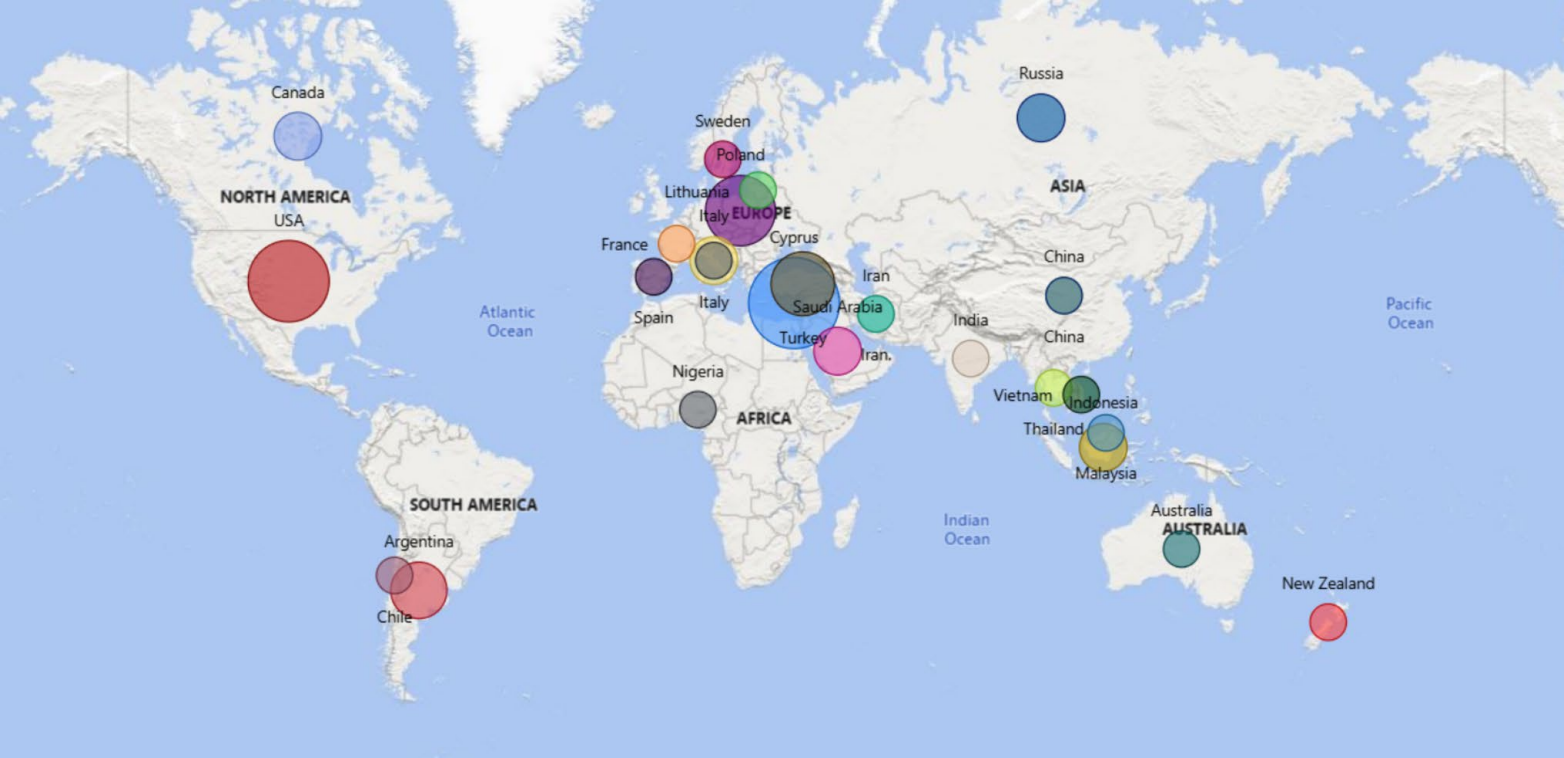
Map visualization shows real bilge sampling locations. Circle size indicates the number of samples. Cyprus accounted for 9 samples, USA 7, Poland 5, Turkey 4, Argentina 3, Canada, Indonesia, Italy, Russia, Saudi Arabia and Sicily all had two and the remaining countries had one

Seventy-seven papers that examined bilgewater characteristics or treatment options were identified and included in this systematic literature review. This included twenty-four studies that characterised the bilgewater from external sites that received and treated bilgewater (Akarsu et al. 2016, Asselin et al. 2008, Bilgili et al. 2016, Carlesi et al. 2015, Corti-Monzón et al. 2021, Emadian et al. 2015b, Gatidou et al. 2022a, Gatidou et al. 2022b, Gatidou et al. 2022c, Gryta 2020a, b, Karakulski and Gryta 2017, Kliaugaite et al. 2006, Mancini et al. 2012, Mazioti et al. 2021b, Mazioti and Vyrides 2024, Mustapha et al. 2021, Nievas et al. 2006, Nikitin et al. 1982, Nisenbaum et al. 2020, Öz and Çetin 2021, Page et al. 2021, Tan et al. 2022, Tomczak and Gryta 2021, Ulucan and Kurt 2015, Vyrides et al. 2018). Twenty-three studies characterised bilgewater samples from maritime vessels including ships (Ameen and Al-Homaidan 2023a, Ameen and Al-Homaidan 2023b, Awel and Fuad 2023, Emadian et al. 2015a; Mazioti et al. 2021a; Olivera et al. 2003; Rizzo et al. 2021; Tomaszewska et al. 2005; Wood et al. 2021), trade ships (Mazioti et al. 2021a), cargo ships (Emadian et al. 2015b; Olorunfemi et al. 2015), passenger ships (Tiselius and Magnusson 2017), fishing vessels (Chanthamalee et al. 2013; Corti-Monzón et al. 2021; Mahendhran et al. 2018; Nisenbaum et al. 2020), cruise ships (Fontana et al. 2023), yachts (Fletcher et al. 2017), research vessels (Schaerer et al. 2021), dredge ships (Corti-Monzón et al. 2021; Nisenbaum et al. 2020), bulk carriers (Shi et al. 2022), container ships (McLaughlin et al. 2014), naval ships (Cazoir et al. 2012; Williams et al. 2007), recreational boats (Fletcher et al. 2017; Maggio et al. 2023; Schaerer et al. 2021) and tugboats (Nunez et al. 2023). Twenty-four studies utilised real bilge samples or synthetic bilge (Akbarzadeh Yazdi et al. 2024, Alper 2005, Church et al. 2021, Corti-Monzon et al. 2020, Diaz et al. 2021, Fiorati et al. 2020, Gentile et al. 2016, Hamidi et al. 2021, Hwang et al. 2021, Körbahti and Artut 2010, 2013, Nguyen et al. 2021, Shen et al. 2020, Son et al. 2019, Sun et al. 2010b, a) to investigate water treatment options. Studies were from a range of countries including Argentina, Australia, Canada, Chile, China, Cyprus, France, India, Indonesia, Iran, Italy, Lithuania, Malaysia, New Zealand, Patagonia, Poland, Russia, Saudi Arabia, Spain, Sweden, Thailand, Turkey, USA and Vietnam Fig. 2.

## Bilgewater parameters

The physicochemical profile of bilgewater is defined by parameters including pH, electrical conductivity, suspended solids, and total dissolved solids (TDS). High conductivity levels, when combined with the presence of aggressive ions such as chloride, sulfate, and bromide, create an environment conducive to electrochemical corrosion (Nunez et al. 2023). Over time, these factors have the potential to impact the ship’s hull and piping systems, representing a relevant factor in vessel maintenance and structural preservation.

From an environmental perspective, the chemical composition of bilgewater suggests a capacity to impact marine ecosystems. The high organic load (TPH, Oil and Grease, COD, and BOD) represents a source of oxygen demand which, under certain conditions, could affect local water quality. Additionally, the presence of nutrient pollutants such as nitrate, ammonium, total nitrogen, and phosphorous indicates a potential pathway for stimulating localized eutrophication and algal growth.

Although this review identified no studies directly quantifying health impacts on shipboard personnel, the chemical profile of bilgewater suggests a potential occupational safety concern. The detection of constituents with established toxicity (Olorunfemi et al. 2015; Özkaynak et al. 2022; Williams et al. 2007) (such as arsenic, cadmium, lead, mercury, chromium, and cyanide) in bilgewater highlights the importance of minimising exposure during maintenance operations. Furthermore, these contaminants remain a concern for the marine environment due to their potential for bioaccumulation in the food web.

The stagnant, nutrient-rich environment of the bilgewater can support the proliferation of a complex microbial community (Nunez et al. 2023), which has the potential to influence the wastewater’s chemical profile and toxicity. This microbiome is often characterised by three functional groups: sulfate-reducing bacteria (SRB), which are associated with microbiologically influenced corrosion; hydrocarbon-degrading bacteria capable of utilising oil and grease constituents; and opportunistic pathogens, which may be introduced via pathways such as port water or sanitary system cross-contamination.

### Chemical characteristics

Twenty-six studies reported the chemical characteristics of bilgewater sampled directly from vessels. As shown in Table 1, these studies demonstrated that the chemical characteristics of bilgewater are highly variable across all categories tested. The pH was the most commonly measured chemical characteristic with 10 papers reporting the pH of samples, followed by chemical oxygen demand (COD) and electrical conductivity with 5 publications each. The reported bilgewater pH varied from 4.85 to 9.0, COD from 20 to 2111 mg/L and electrical conductivity ranged from 1400 to 86500 μS/cm. The analytical methods used for each of the parameters also varied highly between studies. This highlights the need for standardised guidance for characterising bilgewater to determine the environmental impacts and compare treatment options.

**Table 1:** Chemical characteristics of bilgewater sampled from real bilges

Parameter	Range	Number of papers	References
COD_(mg/L)_	20–2111	5	(Ameen and Al-Homaidan 2023a, Corti-Monzón et al. 2021; Emadian et al. 2015a; Mazioti et al. 2021a; Shi et al. 2022)
pH	4.85–9.0	10	(Awel and Fuad 2023, Cazoir et al. 2012; Chanthamalee et al. 2013; Corti-Monzón et al. 2021; Emadian et al. 2015a; Mazioti et al. 2021a; Nisenbaum et al. 2020; Nunez et al. 2023; Olorunfemi et al. 2015; Shi et al. 2022)
Electrical conductivity_(μS/cm)_	1400–86,500	5	(Corti-Monzón et al. 2021; Nunez et al. 2023; Olorunfemi et al. 2015; Shi et al. 2022; Tomaszewska et al. 2005)
BOD_(mg/L)_	6.2–2932	3	(Ameen and Al-Homaidan 2023a, Corti-Monzón et al. 2021; Olorunfemi et al. 2015)
Suspended solids_(mg/L)_	18.4–1840	4	(Chanthamalee et al. 2013; Emadian et al. 2015a; McLaughlin et al. 2014; Tomaszewska et al. 2005)
Total dissolved solids_(mg/L)_	32.5–1307	3	(Awel and Fuad 2023, Olorunfemi et al. 2015; Tomaszewska et al. 2005)
Nitrate_(mg/L)_	99.5	1	(Olorunfemi et al. 2015)
NH_4(mg/L)_	0.17–15	2	(Olorunfemi et al. 2015; Williams et al. 2007)
Total Nitrogen_(mg/L)_	0.5–13.6	3	(Chanthamalee et al. 2013; Emadian et al. 2015a; Williams et al. 2007)
Phosphorous_(mg/L)_	0.211–3.24	3	(Chanthamalee et al. 2013; Emadian et al. 2015a; Williams et al. 2007)
Total petroleum hydrocarbons_(mg/L)_	107–976	4 (a)	(Ameen and Al-Homaidan 2023a, Chanthamalee et al. 2013; Tomaszewska et al. 2005; Williams et al. 2007)
Chromium_(mg/L)_	1.4–69	2	(Fontana et al. 2023; Olorunfemi et al. 2015)
Mercury_(ng/L)_	32.05–79.8	1	(Williams et al. 2007)
Copper_(mg/L)_	0.27–26.8	4	(Ameen and Al-Homaidan 2023a, Fontana et al. 2023; Olorunfemi et al. 2015; Williams et al. 2007)
Iron_(mg/L)_	5.7	1	(Olorunfemi et al. 2015)
Nickel _(mg/L)_	0.245–40	3	(Fontana et al. 2023; Olorunfemi et al. 2015; Williams et al. 2007)
Zinc_(mg/L)_	0.514–20.0	3	(Fontana et al. 2023; Olorunfemi et al. 2015; Williams et al. 2007)
Cadmium_(mg/L)_	0.1	1	(Olorunfemi et al. 2015)
Silver_(mg/L)_	0.2	1	(Olorunfemi et al. 2015)
Potassium_(mg/L)_	184–720	2	(Cazoir et al. 2012; Nunez et al. 2023)
Lead_(mg/L)_	0.3–29.8	3	(Ameen and Al-Homaidan 2023a, Fontana et al. 2023; Olorunfemi et al. 2015)
Manganese_(mg/L)_	3.9–25.4	3	(Ameen and Al-Homaidan 2023a, Fontana et al. 2023; Olorunfemi et al. 2015)
Aluminum_(mg/L)_	54	1	(Fontana et al. 2023)
Chlorine_(mg/L)_	970.3	1	(Olorunfemi et al. 2015)
Sulfate_(mg/L)_	107.5–4927	4	(Cazoir et al. 2012; Nunez et al. 2023; Olorunfemi et al. 2015; Tomaszewska et al. 2005)
Bromide_(mg/L)_	17–157	1	(Nunez et al. 2023)
Magnesium_(mg/L)_	468–2132	2	(Cazoir et al. 2012; Nunez et al. 2023)
Calcium_(mg/L)_	101.52–637	3	(Cazoir et al. 2012; Nunez et al. 2023; Tomaszewska et al. 2005)

^a^Two studies sampled the bilgewater oily phase and thus have a higher TPH of 408,000–1,540,000 mg/L (Corti-Monzón et al. 2021; Nisenbaum et al. 2020)

When considering the environmental impact of the chemical components of bilgewater, hydrocarbons, heavy metals (Fiorati et al. 2020; Olorunfemi et al. 2015), and surfactants (Tiselius and Magnusson 2017) are a cause for concern. Interestingly, although surfactants are required to be added to the IMO synthetic bilgewater cocktails used to measure the efficacy of different OWS, there were no studies that measured the surfactant concentration from authentic bilgewater.

### Biological characteristics

Eleven papers investigated the microbiological communities present in bilgewater samples. Ten of these used 16S rRNA sequencing to analyse the bacterial communities present (Cappello et al. 2012, Corti-Monzón et al. 2021, Gatidou et al. 2022b, Mazioti et al. 2021a, Mazioti and Vyrides 2024, Nisenbaum et al. 2020, Nunez et al. 2023, Olivera et al. 2003, Rizzo et al. 2021, Schaerer et al. 2021, Wood et al. 2021). Of these, six studies focused on bacteria found in the bilgewater that could be used to treat the water and break down hydrocarbons (Cappello et al. 2012, Gatidou et al. 2022b, Mazioti et al. 2021a, Mazioti and Vyrides 2024, Nisenbaum et al. 2020, Olivera et al. 2003, Rizzo et al. 2021). One additional study also used traditional culture and microscopy-based methods to characterise the species present that could break down hydrocarbons. Three studies focused on the diversity of the bacterial communities between sites and sampling events (Corti-Monzón et al. 2021; Olivera et al. 2003; Schaerer et al. 2021) and two studies focused on microbially influenced corrosion (MIC) (Nunez et al. 2023; Wood et al. 2021).

Four studies identified eukaryotes in bilgewater using a combination of methods: eDNA metabarcoding of 18S rRNA or cytochrome oxidase subunit I (COI), traditional microscopy and morphological analysis (Darbyson et al. 2009; Fletcher et al. 2017; Johnson et al. 2001; Maggio et al. 2023). These studies also explored the potential for bilgewater to transfer non-indigenous species. These non-indigenous marine species are primarily transported by ships (Fletcher et al. 2017) and can cause significant biodiversity loss (Maggio et al. 2023). Furthermore, it was shown that there is a potential to transfer invasive species, and this poses a non-negligible biosecurity threat (Darbyson et al. 2009; Fletcher et al. 2017; Johnson et al. 2001; Maggio et al. 2023). No studies investigated the presence of pathogenic or indicator bacteria typically used to assess water quality in healthcare or agriculture. Investigating pathogen levels in bilgewater is crucial, as exposure to contaminated water could pose significant health risks to crew members, potentially leading to gastrointestinal and infectious diseases.

From the 10 studies that utilised 16S rRNA sequencing, *Pseudomonas* followed by *Marinobacter* and *Flavobacterium* were the most frequently detected bacterial species in 7/10, 6/10 and 6/10 studies, respectively. However, bacterial communities varied widely between samples and as shown by Mazioti and co-workers (Mazioti et al. 2021a), the composition of the bacterial community quickly changed following a change in the salinity or COD. Fletcher et al. found that amongst the large variation seen in their samples, there was a core group of hydrocarbon-degrading bacteria able to survive and thrive in the complex bilgewater (Fletcher et al. 2017). Fletcher et al. also identified the core microbiome to consist of *Marinobacter*, which was also found by (Cappello et al. 2012, Gatidou et al. 2022b, Mazioti et al. 2021a, Mazioti and Vyrides 2024, Nisenbaum et al. 2020, Rizzo et al. 2021), *Betaproteobacteria* (Corti-Monzón et al. 2021, Mazioti et al. 2021a, Mazioti and Vyrides 2024, Nisenbaum et al. 2020), bacteria belonging to *Thalassospira* (Corti-Monzón et al. 2021, Mazioti and Vyrides 2024, Nisenbaum et al. 2020), *Parvibaculum* (Gatidou et al. 2022b, Mazioti and Vyrides 2024, Nisenbaum et al. 2020), and *Alcanivorax* (Gatidou et al. 2022b, Mazioti and Vyrides 2024, Nisenbaum et al. 2020, Rizzo et al. 2021) and *Pseudomonas* (Cappello et al. 2012, Corti-Monzón et al. 2021, Mazioti and Vyrides 2024, Nisenbaum et al. 2020, Nunez et al. 2023, Olivera et al. 2003, Rizzo et al. 2021) and undescribed genus/species of the *Flavobacteriaceae* (Corti-Monzón et al. 2021, Gatidou et al. 2022b, Mazioti et al. 2021a, Mazioti and Vyrides 2024, Nisenbaum et al. 2020, Nunez et al. 2023) and *Rhodospirillaceae* (Nisenbaum et al. 2020) families and the Gammaproteobacterial order known as PYR10d3 (Corti-Monzón et al. 2021; Nisenbaum et al. 2020).

While there were no papers that specifically looked for the presence of pathogenic bacteria, several genera were found that contain species with the potential to be pathogenic to humans. For example, *Desulfovibrio* (Nunez et al. 2023), *Acinetobacter* (Rizzo et al. 2021), *Pseudomonas* (Cappello et al. 2012, Corti-Monzón et al. 2021, Mazioti and Vyrides 2024, Nisenbaum et al. 2020, Nunez et al. 2023, Olivera et al. 2003, Rizzo et al. 2021), *Shewanella putrefaciens* (Olivera et al. 2003) and *Achromobacter* (Nisenbaum et al. 2020) have all been linked to human disease. The bacterial communities found in bilgewater also influence asset management, with MIC presenting a challenge for the shipping industry (Hill and Hill 1994, Wood et al. 2021). MIC can lead to increased maintenance costs and the need for advanced mitigation strategies. It can be caused by sulfate-reducing bacteria (SRB), metal depositing bacteria, or even cell-free extracellular polymeric substances EPS (Knisz et al. 2023). These SRB can cause metal corrosion through the production of acidic by-products; SRB species *Desulfovibrio* have been shown to increase MIC and were detected in (Nunez et al. 2023).

### Treatment options

**Fig. 3:**
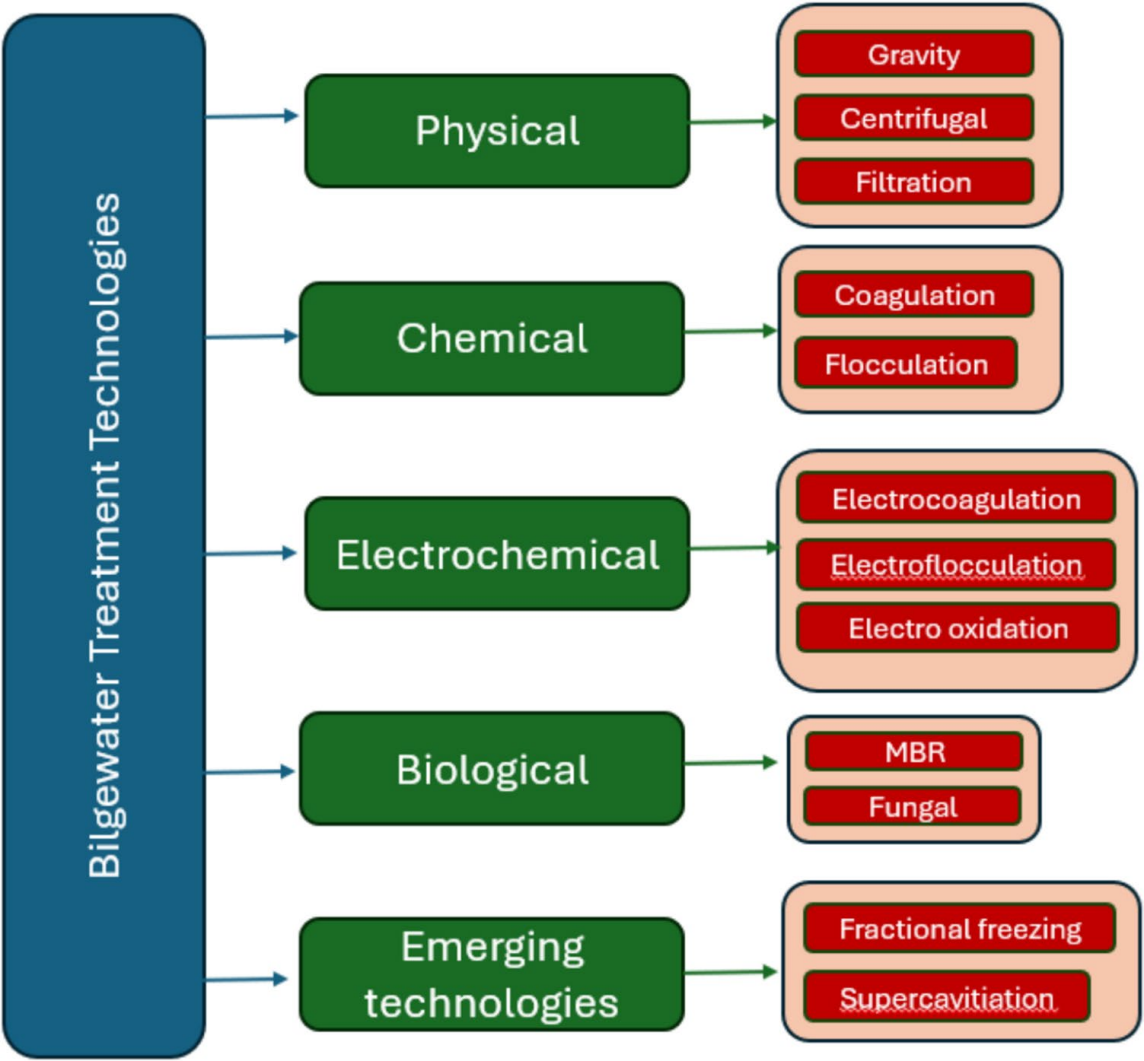
Bilgewater treatment technologies

The primary technology employed to ensure vessel compliance with bilgewater discharge regulations is the Oily Water Separator (OWS). These systems are certified under IMO MEPC.107(49) to verify their ability to reduce effluent oil content below the 15 ppm limit mandated by MARPOL Annex I (Riadh 2024). A wide variety of OWS technologies exist to meet these standards, ranging from gravity-based separation to advanced centrifugal and filtration systems, each possessing distinct operational advantages and limitations. While all certified units must demonstrate performance under simulated operational conditions, the specific mechanisms they employ to achieve separation vary significantly. The highly variable nature of bilgewater also makes its treatment complicated (Fig. 3).

Of the seventy-seven identified papers, fifty-six looked at different approaches to treating bilgewater to meet the MARPOL requirement of less than 15 ppm of oil. Twenty-three used hydrocarbonoclastic bacteria to metabolise the oil (Ameen and Al-Homaidan 2023a, Ameen and Al-Homaidan 2023b, Chanthamalee et al. 2013, Corti-Monzón et al. 2021, Corti-Monzon et al. 2020, Emadian et al. 2015a, Emadian et al. 2015b, Gatidou et al. 2022b, Gentile et al. 2016, Mahendhran et al. 2018, Mancini et al. 2012, Mazioti et al. 2021a, Mazioti et al. 2020, Mazioti et al. 2021b, Mazioti and Vyrides 2024, Nievas et al. 2006, Nisenbaum et al. 2020, Olivera et al. 2003, Rizzo et al. 2021, Shi et al. 2022, Sun et al. 2010b, a, Vyrides et al. 2018). Four used electrochemical treatment (Carlesi et al. 2015, Körbahti and Artut 2010, 2013, Ulucan and Kurt 2015). Five used filtration (Gryta 2020a, 2020b, Karakulski and Gryta 2017, McLaughlin et al. 2014, Sun et al. 2010a, Tan et al. 2022, Tomczak & Gryta 2021). Asselin et al. (2008), Bilgili et al. (2016), and Soeprijanto et al. (2019) utilised electrocoagulation, while Hamidi et al. (2021) employed a coagulation-flocculation process. Two used oxidation (Akbarzadeh Yazdi et al. 2024; Fontana et al. 2023) and one each focused on flocculation (Beryoza et al. 2021), supercavitation (Malakhov et al. 2024), biochemical (Nikitin et al. 1982) and adsorption (Kliaugaite et al. 2006). There were seven that focused on using multistage systems (Akarsu et al. 2016, Cazoir et al. 2012, Gatidou et al. 2022a, Gatidou et al. 2022c, Karakulski and Gryta 2017, Nguyen et al. 2021, Öz and Çetin 2021). McLaughlin et al. (2014) characterised bilgewater before and after treatment in 3 different systems.

The variation in the composition of the bilgewater, starting conditions, run times, and chemical components measured makes it challenging to compare the efficacy of the treatment options across studies. However, COD reduction rate was the most frequently reported parameter for evaluating efficacy (Table 2).

**Table 2:** Chemical oxygen demand (COD) removal efficiency of treatment methods

Treatment method	COD reduction %	References
Biological	28–83%	(Emadian et al. 2015b, Gatidou et al. 2022b, Gatidou et al. 2022c, Mancini et al. 2012, Mazioti et al. 2021b, Mazioti and Vyrides 2024, Shi et al. 2022, Vyrides et al. 2018)
Electrochemical	37.5–90.1%	(Körbahti and Artut 2013)
Coagulation	92%	(Hamidi et al. 2021)
Electrocoagulation	61.3–78.1%	(Akarsu et al. 2016; Asselin et al. 2008, Ulucan and Kurt 2015)
Oxidation	89.5%	(Öz and Çetin 2021)
Adsorption	86.7%	(Kliaugaite et al. 2006)

#### Physical treatment methods

Physical treatment technologies rely primarily on density difference, particle size, and surface adhesion to separate dispersed oil and solids from the bilgewater.

##### Gravity separation

This method takes advantage of the density difference between oil and water to separate the two phases. It is commonly used as a primary treatment step to separate large oil droplets from the water column. While effective for free oil, gravity separation faces significant limitations in the maritime environment. The presence of surfactants, engine vibrations, and vessel movement, oil and water often results in the formation of stable emulsions that are difficult to separate via gravity alone (Diaz et al. 2021). Furthermore, effective separation often requires long settling times, which may be impractical for shipboard operations (Williams et al. 2007).

##### Centrifugal separation

Centrifugal separators also take advantage of density differences between oil and water, but accelerate the rate of separation by rapidly spinning the bilgewater, achieving forces up to 8000 G. This technology offers a solution for mechanically emulsified fluids; however, its inability to process large volumes of bilgewater in short periods (Malakhov et al. 2024) remains a primary disadvantage.

##### Coalescing plate separators

These systems use closely spaced parallel plates to enhance gravity separation. The plates provide a surface for small oil droplets to collide, coalesce into larger droplets, and rise to the surface for removal.

Coalescing plates improve the efficiency of standard gravity separation but remain susceptible to fouling and reduced efficiency when handling chemically stabilised emulsions.

#### Filtration and membrane processes

Filtration technologies separate contaminants based on size exclusion and, in some cases, adsorption. These processes range from coarse media filtration to molecular-scale membrane separation. Three main membrane filtration technologies have been reported.

***Microfiltration (MF)*** utilises pore sizes in the range of 0.1 to 10 µm. It is primarily designed to remove suspended solids and large emulsified oil droplets. Alternately, with pore sizes in the range of 0.01–0.1 microns.

***Ultrafiltration (UF)*** is capable of removing finer emulsions and macromolecules. Utilising semi-permeable membranes that block dissolved ions,

***Reverse osmosis (RO)*** is employed when the objective is to remove dissolved salts and metals in addition to hydrocarbons.

Filtration is an effective method for removing oil from bilgewater, capable of achieving concentrations significantly below the 15 ppm MARPOL limit (Karakulski & Gryta 2017, McLaughlin et al. 2014; Sun et al. 2010a, Tan et al. 2022) and reducing other pollutants (McLaughlin et al. 2014). Various membrane materials are used, including ceramic (Sun et al. 2010a, Tomczak and Gryta 2021), polyvinylidene fluoride (PVDF) (Karakulski and Gryta 2017), piezoelectric composites (BaTiO3/PVDF) (Tan et al. 2022), and polypropylene (Gryta 2020b).

However, filtration performance is often hindered by membrane fouling from suspended solids or the build-up of CaCO_3_. This necessitates mitigation strategies such as bilgewater pretreatment (e.g., with biofilm-membrane bioreactors) (Sun et al. 2010a) and regular filter cleaning. Modifications such as using polypropylene membranes, which resist wetting (Gryta 2020b), or employing vibrating piezoelectric membranes (Tan et al. 2022), can reduce fouling rates. Common cleaning methods include periodic rinsing with HCl solution (Gryta 2020a, b) or alkaline agents (Karakulski and Gryta 2017) to remove precipitates, or using solutions like 1–3% NaOH or H_3_PO_4_. While cleaning can restore permeate flux, the required downtime and cost can be prohibitive. Consequently, filtration, including ultrafiltration or reverse osmosis, is often employed most effectively as a final polishing step within a multi-barrier treatment approach after initial turbidity and COD reduction (Karakulski and Gryta 2017).

#### Chemical treatment technologies

##### Chemical coagulation

Chemical coagulation involves the addition of chemical agents to destabilise charged particles suspended in the bilgewater, allowing them to aggregate. This can be a low-cost and compact method of treatment suitable for use onboard ships (Hamidi et al. 2021). However, it is used less frequently than other methods due to the complexity of bilgewater composition, high operational costs, and the potential for secondary pollution from chemical additives (Medeiros et al. 2022). While the environmental cost of inorganic coagulants can be high, natural coagulants like tuber starch or red lentil extract have been shown to be effective alternatives. Specifically, Orchis mascula tubers were investigated as a biodegradable, non-toxic coagulant source (Hamidi et al. 2021).

##### Flocculation

Flocculation typically follows coagulation. The water is subjected to gentle mixing, often with the addition of a long-chain polymer (flocculant). These molecules bridge neutralised micro-particles, aggregating them into large, visible “flocs” that can be easily separated via sedimentation or filtration (Medeiros et al. 2022). Flocculation can be used in conjunction with coagulation to improve the purification process (Hamidi et al. 2021). However, (Beryoza et al. 2021) pointed out that this can cause corrosion and a significant amount of sediment. They evaluated several high-molecular-weight flocculants and determined that cationic variants achieved the highest purification efficiency due to their specific ability to neutralise negatively charged emulsified oil particles.

#### Biological treatment methods

##### Hydrocarbon degrading bacteria

Biological treatment utilises hydrocarbon-degrading bacteria, either suspended in a bioreactor or introduced as an additive, to metabolise hydrocarbons and organic contaminants. Biological treatments can make use of either endogenous bacteria (Nievas et al. 2006; Nisenbaum et al. 2020; Vyrides et al. 2018), a specific bacterial consortium (Gatidou et al. 2022b; Mancini et al. 2012), or fungi (Ameen and Al-Homaidan 2023b). These bacteria can adapt to the changing conditions found in bilgewater, with different species dominating the culture depending on the specific pollutants present (Mazioti et al. 2021a). However, the adaptation period for the bacterial community can be significant, and the hydraulic retention times required for effective treatment are often long (Mazioti et al. 2021a; Vyrides et al. 2018), which may present challenges for compact shipboard applications.

##### Membrane bioreactors (MBR)

MBR systems integrate biological degradation with membrane filtration. The membrane is submerged directly into the bioreactor, replacing the settling tank used in conventional activated sludge systems, acting as a barrier to retain biomass while allowing treated water to permeate. While effective, membrane fouling represents a critical operational challenge in these systems (Sun et al. 2010b). Specifically, the accumulation of oil and oily biomass aggregates creates a sticky fouling layer that compromises filtration performance, necessitating rigorous maintenance or pretreatment protocols.

##### Moving bed biofilm reactors (MBBR)

MBBR technology uses circulating plastic carriers within an aerated tank to support biofilm growth (Vyrides et al. 2018). This configuration allows diverse bacterial communities to adhere to the carriers and degrade contaminants within a compact footprint. (Mazioti et al. 2021a) demonstrated that aerobic MBBRs treating highly saline bilgewater could achieve up to 86% COD removal, maintaining operational stability even during significant salinity and organic load shocks.

##### Fungal treatment

This method utilises the potent enzymatic capabilities of fungi to metabolise hydrocarbons. Specific strains are cultivated to degrade the complex organic compounds found in heavy fuels and lubricants, offering a potential alternative to bacterial systems for recalcitrant pollutants (Ameen and Al-Homaidan 2023b).

#### Electrochemical methods

##### Electrocoagulation

Electrocoagulation involves the in-situ generation of coagulants by dissolving sacrificial metal electrodes (usually aluminum or iron) using a direct electrical current. This technology reduces the requirement for additional chemical treatment (Asselin et al. 2008), can be fully automated, and is compact (Bilgili et al. 2016). It is effective against a wide range of contaminants; in addition to treating COD, electrocoagulation can decrease pollutants such as metals and sulfates (Bilgili et al. 2016), turbidity, and BOD (Asselin et al. 2008).

##### Electro-flotation (EF)

EF focuses specifically on the generation of gas bubbles (Hydrogen and Oxygen) via water electrolysis (Ulucan and Kurt 2015). These bubbles are extremely fine and uniform, allowing them to collide more frequently with small oil droplets and float them to the surface for removal (Akbarzadeh Yazdi et al. 2024). Electroflotation is rarely used alone; it usually works in tandem with electrocoagulation or electro-oxidation (Asselin et al. 2008, Ulucan and Kurt 2015). This creates a synergistic effect where destabilised emulsions and flocculated particles are physically lifted and separated from the aqueous phase. While the compact footprint of these systems makes them ideal for shipboard applications, their efficiency is highly time-dependent (Akbarzadeh Yazdi et al. 2024). Success relies on fine-tuning the current density (Bian et al. 2019) to ensure there is enough bubble-particle collision and bonding to actually clear the water.

##### Electro-oxidation (EO)

Electro-oxidation is an advanced oxidation process that degrades organic pollutants through direct or indirect oxidation at the anode surface, effectively mineralising contaminants rather than just separating them (Ulucan and Kurt 2015). This approach is particularly effective for hitting strict COD discharge limits since it targets dissolved organics without generating the heavy sludge associated with other methods (Akbarzadeh Yazdi et al. 2024). However, it does require high-spec, corrosion-resistant electrodes like platinum or boron-doped diamond (BDD) (Akbarzadeh Yazdi et al. 2024, Körbahti and Artut 2010, 2013). While the efficiency is high, the real-world trade-offs are the heavy energy demand and the risk of forming chlorinated by-products or stable chlorates (Körbahti and Artut 2013), especially if the pH is not tightly controlled.

#### Emerging technologies

##### Supercavitation

This process involves passing fluid through a constriction (like a venturi) or using a rotating generator to increase fluid velocity, causing local static pressure to drop below the vapor pressure. This forms bubbles that collapse violently downstream, generating localized hot spots, shear forces, and shock waves. Malakhov et al. (2024) propose this technology to overcome the “unidirectional” limitations of traditional physical and chemical separators, which purify water but fail to recover useful hydrocarbons.

##### Fractional freezing

Fractional freezing operates on the principle that the crystal lattice of ice naturally excludes dissolved solutes and impurities during formation, concentrating the pollutants in the remaining liquid phase (Mustapha et al. 2021). The success of this physical separation depends heavily on tight control over coolant temperature (Mustapha et al. 2021) and freezing time to prevent oil impurities from being “trapped” within the forming ice lattice. When optimised, specifically through progressive fractional freezing (PFF) with proper stirring, the process can bring oil content down to the 15ppm limit required by international discharge standards.

#### Challenges and integrated approaches

One of the primary challenges identified in this review is that the oil in bilgewater consists of both a gravity-separable phase and a chemically stabilised emulsified phase. Consequently, treatment options must be designed to address both. This necessitates the use of multistage or hybrid treatment options to ensure treated water meets MARPOL discharge regulations.

Furthermore, while invasive species have been confirmed to exist in bilgewater (Fletcher et al. 2021; Maggio et al. 2023), and treatment protocols are established for ballast water, no studies identified in this search have evaluated the efficacy of current bilgewater treatment technologies against biological threats specifically within the complex chemical matrix of bilgewater.

## Discussion

### Regulatory adequacy

The findings of this systematic literature review underscore the complex and variable nature of bilgewater and highlight the potential for negative effects on the environment associated with its discharge. While current regulations have contributed to a reduction in oil pollution from ships, the presence of other contaminants in bilgewater, including heavy metals, nutrients, and potentially pathogenic microorganisms, raises concerns about the adequacy of existing regulatory frameworks in protecting marine ecosystems. Current regulations under MARPOL Annex I focus specifically on oil content; consequently, they do not address the full spectrum of other contaminants present in bilgewater.

While the MARPOL Convention serves as a global framework for regulating the discharge of ship-sourced pollutants, including bilgewater, signatory nations often implement supplementary regulations that reflect specific regional concerns and environmental sensitivities. This is evidenced by the adoption of more stringent discharge criteria in several jurisdictions. For instance, the European Union, under Directive 2019/883, mandates the use of port reception facilities for ship-generated waste and prohibits illicit discharges of oily residues within European waters, emphasizing a preventative approach to marine pollution (EU 2019). Similarly, Australia’s Protection of the Sea (Prevention of Pollution from Ships) Act 1983 designates specific environmentally sensitive areas, such as the Great Barrier Reef, where bilgewater discharge is strictly prohibited (Commonwealth of Australia 1983). This approach is mirrored in the United States Clean Water Act, which establishes “no discharge zones” to safeguard specific water bodies from the impacts of bilgewater effluents (USEPA 2024). Furthermore, New Zealand’s Maritime Transport Act 1994 extends protection to ecologically significant areas by prohibiting the discharge of even treated bilgewater within their boundaries, underscoring a precautionary principle towards vulnerable marine ecosystems (New Zealand 1994). These examples highlight a growing trend towards the adoption of region-specific regulations that surpass the baseline requirements of MARPOL, reflecting an increasing awareness of the localized impacts of bilgewater and a commitment to enhanced marine environmental protection.

### Ecological risks

As identified by this review, bilgewater discharge from ships may introduce contaminants into marine environments that could pose a potential threat to ecosystem health. Specifically, the review identified elevated concentrations of hydrocarbons, including total petroleum hydrocarbons (TPH) ranging from 6 to 976 mg/L and oil and grease from 6.5 to 900 mg/L. These substances are known to have severe impacts on marine life, causing physical smothering, disrupting physiological processes, and contaminating habitats (Peterson et al. 2003). Furthermore, the analysis found elevated levels of other pollutants in bilgewater, including COD, BOD, nitrogen, and phosphorus. Such concentrations can stimulate excessive growth of algae and microbes, leading to eutrophication in coastal waters (Breitburg et al. 2018; Wei et al. 2021), harmful algal blooms, and subsequent loss of biodiversity. While shipping has been shown to contribute very small amounts of nitrogen (1.25–3.3%) and phosphorus (0.3%) (Raudsepp et al. 2019), these contributions can still be significant in nutrient-sensitive marine ecosystems, where even small increases can exacerbate issues like eutrophication and harmful algal blooms.

The presence of trace metals found in bilgewater may also pose a risk to the environment. This includes concentrations of mercury up to 0.0007 mg/L, cadmium up to 0.2 mg/L (Williams et al. 2007), lead up to 29.8 mg/L (Ameen and Al-Homaidan 2023a) and copper up to 426 mg/L (Williams et al. 2007), which can bioaccumulate in marine organisms, leading to biomagnification up the food chain and potential harm to top predators, including humans (Özkaynak et al. 2022). This literature review identified that bilgewater discharge has the potential to contribute to increased heavy metal concentrations, potentially exceeding toxic thresholds in localized areas (Shah 2021). However, it is important to note that the high dilution capacity of the open ocean is likely to significantly reduce these concentrations post-discharge.

Bilgewater may also contain other contaminants that pose risks to marine ecosystems. Surfactants, for instance, are not currently regulated under MARPOL (Julian 2000), yet their release into the marine environment may cause toxicity to marine wildlife (Tiselius and Magnusson 2017). However, this is an area needing further research as no studies investigated the presence of surfactants in real bilgewater.

Within the regulatory framework governing maritime pollution, the concentration of oil in bilgewater has historically served as the principal metric for compliance and, subsequently, as the primary indicator of treatment efficacy. This is further underscored by the IMO MEPC.107(49) (International Maritime Organization 2003) guidelines for the certification of OWS, which stipulate the use of standardized test fluids intended to mimic real-world bilgewater conditions. However, the present literature review reveals a critical limitation as studies often utilise synthetic bilgewater without presenting direct comparative data against authentic bilgewater, making it difficult to verify if these surrogates are able to adequately capture its complex and dynamic nature. Empirical evidence demonstrates that bilgewater composition is subject to substantial variability, influenced by a multitude of factors including vessel age and maintenance, geographic location of sampling, fuel type employed, and prevailing environmental conditions, encompassing both seasonal and operational fluctuations. Consequently, current protocols relying on synthetic fluids and a single metric likely overestimate treatment reliability while underestimating the environmental risks of real-world discharge.

### Treatment efficacy

The presence of stable oil–water emulsions, common in bilgewater, further complicates treatment processes (Alper 2005). These emulsions, stabilized by surfactants, temperature, and vessel motion, require specialized treatment for effective separation and compliance with discharge limits. Bilgewater with atypical contaminants, such as high chemical concentrations or accumulated sludge and fine particles, can exceed OWS design parameters. This can lead to increased maintenance, reduced efficiency, and potentially frustrating crews, thereby increasing the likelihood of intentional system bypassing or neglect.

Coagulation is one treatment method that shows some potential for the removal of heavy metals (Hamidi et al. 2021). However, there is currently limited knowledge on the effectiveness of bilgewater treatment methods for the removal of other contaminants. The comparison of treatment options is further complicated by the lack of standardised assessment approaches. To support future research, standardised methods to assess bilgewater treatment need to be developed. This should include standardised approaches to sample collection, handling and measurement of contaminants. This would enable comparisons between studies and would support improved control and regulations around marine discharges to safeguard marine ecosystems.

### Knowledge gaps

A significant knowledge gap identified in this literature review is the limited understanding of the relationship and interactions between the chemical composition of bilgewater and its bacterial communities. While we attempted to analyse longitudinal patterns in composition and treatment efficacy, the high heterogeneity of existing studies, stemming from varied vessel types, sampling protocols, and analytical methodologies, precluded the identification of statistically robust trends. Exploring this relationship could provide valuable insights into addressing challenges such as MIC (Knisz et al. 2023; Liu et al. 2023; Nunez et al. 2023), the spread of invasive microbial species (Schaerer et al. 2019), and safeguarding crew well-being by minimising potential exposure to microbial contaminants. While the presence of hydrocarbon-degrading bacteria is important, it does not guarantee active breakdown of those hydrocarbons. Factors like pH, nutrients, oxygen, temperature, and the bacterial communities’ prior adaptation to hydrocarbons all play a role (Leahy and Colwell 1990). Successful biological treatment of bilgewater to IMO standards and beyond requires a comprehensive understanding of these factors. MIC is a complex phenomenon and management requires a detailed knowledge of the ecology found in the bilge as well as functional interactions between the microbial consortia and the environment (Knisz et al. 2023). The chemical composition of the media is one of the crucial foundations for the formation of MIC (Little et al. 2020). The formation of biofilm is a further key factor in MIC, and is triggered by specific environmental cues (Tolker-Nielsen 2015). Once formed, the microbial communities often differ greatly between planktonic and sessile, with the planktonic communities having a much lower impact on MIC (Dockens et al. 2017; Wood et al. 2021; Wrangham and Summer 2013). A deeper understanding of the bilge environment could lead to improved modelling, forecasting, and treatment of MIC and improved management of maritime assets.

### Species transfer

Vessel movements are a recognized pathway for the transfer of marine non-indigenous species (NIS), which pose a theoretical threat to native ecosystems and industries. While regulations effectively target ballast water (Julian 2000), the role of bilgewater as a secondary vector is often overlooked. Bilgewater has been shown to contain the larvae and juveniles of invertebrates (Fletcher et al. 2017), suggesting it transports more than just microbes. While acknowledging potential methodological biases, researchers conclude that bilgewater remains a plausible vector for the introduction of these species (Maggio et al. 2023). Consequently, a deeper understanding of bilgewater composition is required to evaluate the survival potential of these organisms and the associated risks of transfer.

### Human health risks

This systematic literature review found there is limited research investigating the potential for bilgewater to pose a risk to human health. The presence of pathogenic bacteria genera such as *Pseudomonas*, *Staphylococcus* and *Escherichia* (Schaerer et al. 2019) indicates that pathogenic bacteria may be present. Bilgewater has also been implicated in the spread of cholera (McCarthy and Khambaty 1994). As sailors can be obliged to work in the bilges, future research should determine the potential risk and identify appropriate management and risk mitigation strategies to protect sailors.

### Consequences of inadequate bilgewater treatment

Beyond the technical challenges of managing microbial consortia, the failure to effectively treat bilgewater carries profound risks. Illegal discharge results in environmental degradation and the erosion of public trust, while MARPOL-driven enforcement mechanisms create significant legal liabilities. Economically, non-compliance gives an unfair competitive advantage to bad actors. These risks are compounded by systemic inequities, as financial constraints often prevent smaller operators from accessing advanced treatment technologies, leaving resource-poor regions disproportionately vulnerable to persistent contamination.

## Conclusion

This systematic literature review on bilgewater characterisation revealed high variability in its chemical and microbial composition, significantly impacting treatment technology efficacy. Current IMO regulations only limit oil content in discharged bilgewater, neglecting other contaminants and leading to a research gap regarding the effectiveness of treatment technologies beyond oil removal. Moreover, the reliance on simplistic synthetic bilgewater cocktails for OWS certification fails to represent the complexity and variability of real bilgewater. Therefore, future research should focus on standardising bilgewater characterisation methods to assess the environmental and asset-related impacts of both untreated and treated bilgewater. Developing integrated treatment systems capable of addressing the full spectrum of contaminants is also crucial. Implementing more rigorous testing requirements that consider bilgewater variability would enhance bilgewater treatment system design and performance. Addressing these challenges is critical to mitigating the risks associated with bilgewater discharge, supporting the development of improved regulations and management practices, and ultimately ensuring better protection of our marine ecosystems.

## Supplementary Information

Below is the link to the electronic supplementary material.

Supplementary file1 (XLSX 28 KB)

## Data Availability

The authors declare that the data supporting the findings of this study are available within the paper.
